# Pulmonary Metastases of Chondroblastoma in a Pediatric Patient: A Case Report and Review of Literature

**DOI:** 10.7759/cureus.28897

**Published:** 2022-09-07

**Authors:** Courtney Wing, Pankaj Watal, Monica Epelman, Juan Infante, Tushar Chandra

**Affiliations:** 1 Pediatric Radiology, Nemours Children's Hospital/University of Central Florida College of Medicine, Orlando, USA

**Keywords:** chondroblastoma, pediatrics, metastases, lung, denosumab, recurrent chondroblastoma, pulmonary metastases of chondroblastoma, pediatric chondroblastoma

## Abstract

Chondroblastoma is a locally destructive, cartilaginous bone tumor that accounts for a small percentage of cases of primary bone tumors. Although considered a benign tumor, chondroblastoma can locally recur and can rarely metastasize. Here, we report a rare presentation of a locally recurrent chondroblastoma with pulmonary metastases. A 13-year-old female presented with palpitations, dry cough, difficulty breathing, and chest tightness four years after her original surgical resection of tibial chondroblastoma. On chest CT, multiple pulmonary soft tissue nodules with confluent punctate areas of calcification were seen. The patient underwent robotic-assisted bilateral pulmonary wedge resections. She is now undergoing denosumab therapy. This case underlined the importance of suspecting metastatic disease in patients with a history of chondroblastoma when pulmonary nodules are detected on imaging.

## Introduction

Chondroblastoma is a locally destructive, rare cartilaginous bone tumor that accounts for about 1% of cases of primary bone tumors. It is most commonly found in patients aged 10-20 years with a male to female ratio of 2:1. The primary tumor arises from the epiphysis of a long bone, with the most common locations being the proximal femur, proximal tibia, distal femur, and proximal humerus [1]. Although it is considered a benign tumor, chondroblastoma can recur locally, is locally destructive, and rarely metastasize [1]. It is known to metastasize to the lung, bone, and soft tissue. Of these sites, it mainly metastasizes in the lung [2]. Here, we report a case of locally recurrent pediatric epiphyseal chondroblastoma with pulmonary metastases. 

## Case presentation

**Figure 1:**
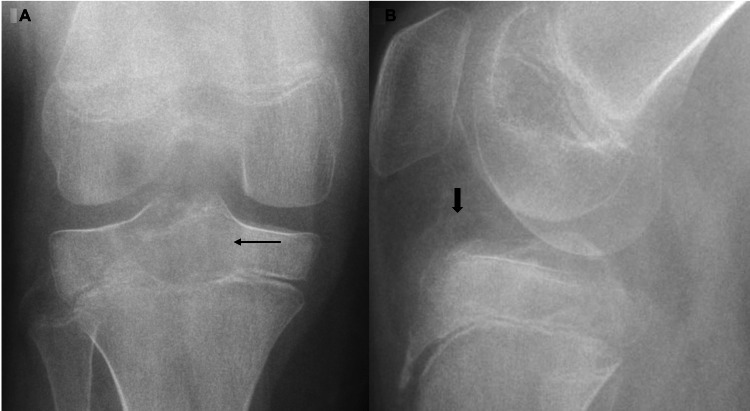
Frontal (A) and lateral (B) radiographs of right knee. Focal lucency within central proximal tibia epiphysis (thin arrow) with suspected ill-defined areas of loose calcification (thick arrow) in anterior intercondylar space. The overall appearance is suspicious for a focal mass centered in the epiphysis.

A nine-year-old Caucasian female presented to our institute with a history of pain in right knee for five months. The patient’s past medical history was significant for frequent upper respiratory tract infections, asthma, gastroesophageal reflux disease (GERD), and neonatal abstinence syndrome. The patient had no prior surgical history upon presentation. Plain radiograph of the right knee revealed a focal lytic lesion in the proximal right tibial epiphysis (Figures 1A, 1B).

**Figure 2:**
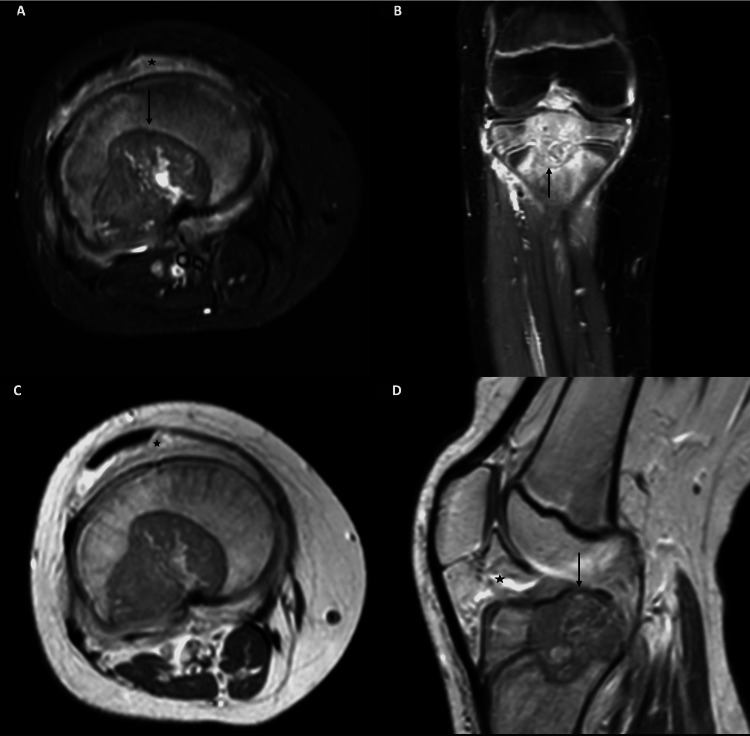
MRI knee at presentation with precontrast T2-weighted image (A) and postgadolinium contrast images in coronal (B), axial (C), and sagittal (D) planes. A defined lobular appearing heterogeneous signal intensity lesion (arrow) (A) is identified centered in the central portion of the proximal tibial epiphysis (arrow) (B). The mass extends across the proximal tibial physis into the adjacent metaphysis (arrow) (B and D). Postcontrast imaging shows diffuse heterogeneous enhancement. Note diffuse signal changes and enhancement in the surrounding marrow, periosteal soft issues, and knee joint space (asterisk), which is probably reactive.

MRI of the right knee demonstrated a focal lesion centered in the right tibial epiphysis and revealed extension into the central and posterolateral proximal tibial physis and proximal tibial metaphysis with diffuse heterogeneous enhancement (Figures 2A-2D). These findings were highly suspicious of chondroblastoma.

One month later, the patient underwent surgery and bone grafting. The microscopic description of the excised tissue described a tumor composed of mononuclear cells with grooved nuclei, “chicken wire calcifications” surrounding the cells, foci with chondroid appearing matrix, scattered multinucleated giant cells, and focal necrosis. The final diagnosis of chondroblastoma was made based on pathology.

**Figure 3:**
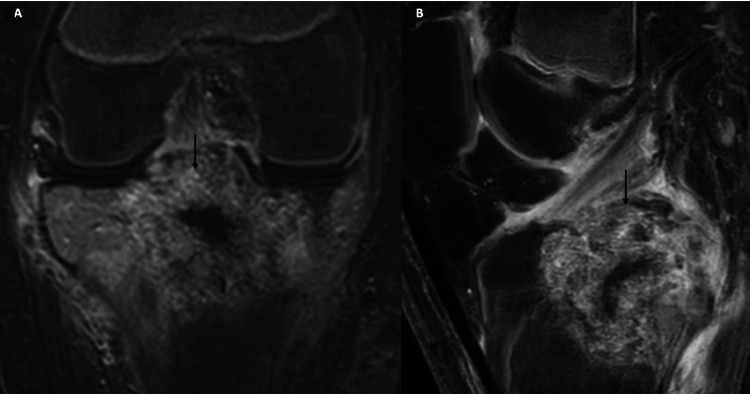
MRI knee postgadolinium contrast images in coronal (A) and sagittal (B) planes at follow-up of six months. Postcontrast imaging shows a markedly heterogeneous enhancing mass in the proximal tibial epiphysis, also involving the adjacent metaphysis across the physis (arrow). This likely represents tumor recurrence in the given clinical setting.

Six months after the original tumor resection, a follow-up MRI of the knee revealed a suspicious mass involving the proximal right tibial epiphysis, physis, and metaphysis. Imaging was also suggestive of edema in the adjacent proximal tibial epiphysis and diaphysis (Figures 3A, 3B). This was highly suggestive of recurrent tumor. The patient was again taken to surgery and the lesion was resected. The tumor material was sent for a frozen section, and the cavity was filled with bone cement (polymethyl methacrylate). Pathology of the excised lesion demonstrated “tumor cells similar to those seen in the previous case" (mononuclear cells with grooved nuclei, “chicken wire calcifications” surrounding the cells, foci with chondroid appearing matrix, scattered multinucleated giant cells) and a diagnosis of recurrent chondroblastoma was made. No atypical mitoses were observed.

**Figure 4:**
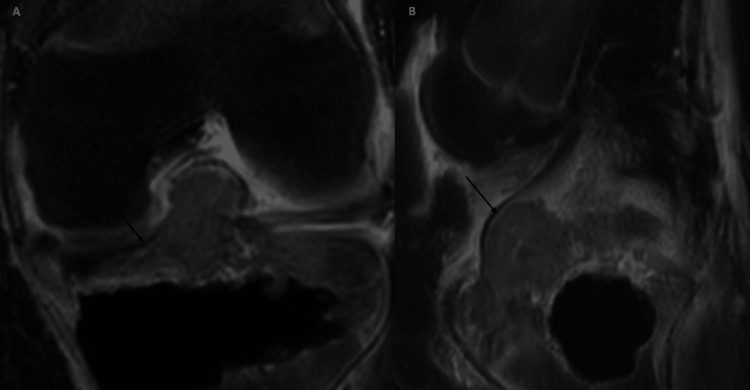
MRI knee postgadolinium contrast images in coronal (A) and sagittal (B) planes at follow-up of 10 months. Postcontrast imaging shows interval progression of heterogeneous enhancing mass with further extension into the knee joint space (arrow). The mass now shows intra-articular extension into the knee joint with mass effect upon the anterior cruciate ligament.

Negative tumor margins were not achieved; five months later, the patient was again diagnosed with recurrent chondroblastoma on a surveillance MRI (Figures 4A, 4B). A recurrent tumor was detected with intra-articular extension into the knee joint with mass effect upon the anterior cruciate ligament. Therefore radical resection of the right tibia and total knee arthroplasty was indicated for the patient and was performed. 

**Figure 5:**
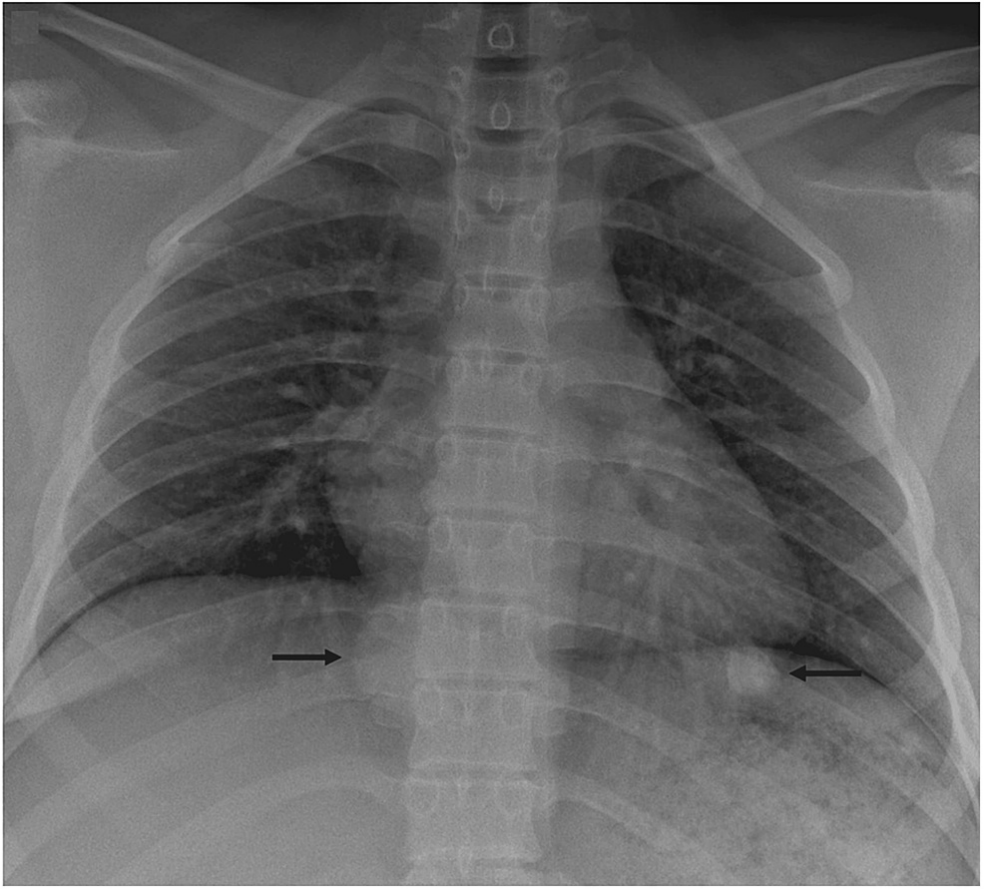
Radiograph of chest at follow-up of 4.5 years. Two defined dense nodules are identified in right and left lower lung (arrows).

Three years after radical resection and arthroplasty of the right knee, the patient presented for evaluation of palpitations with reported symptoms of dry cough, difficulty breathing, and chest tightness. Electrocardiogram and echocardiogram were insignificant on evaluation. On chest radiograph, a well-defined round nodule was seen over the left lower lobe, as well as a dome shaped opacity in the left paraspinal region of the lung (Figure 5). 

**Figure 6:**
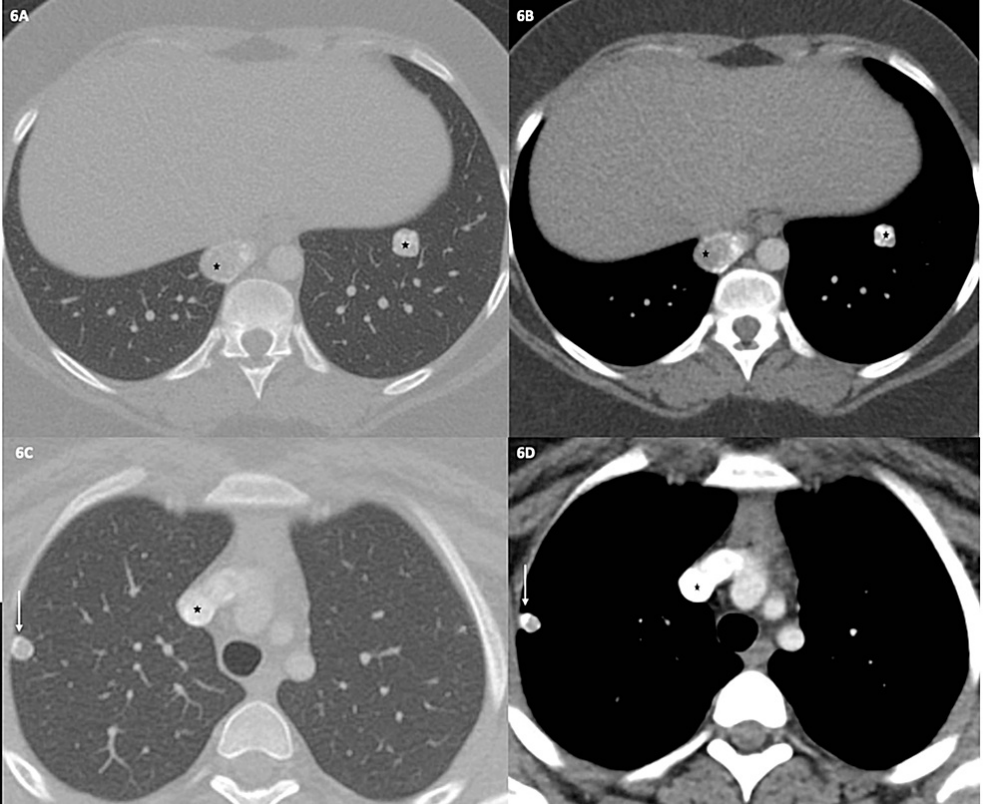
Chest CT in lung and mediastinal windows at two levels (A-D). Chest CT at lower level (A and B) confirm the nodules in prior chest radiograph (asterisks). The paramediastinal nodule in the right lower lobe shows central soft-tissue attenuation with a peripheral rim of calcification (asterisk) (C and D). The nodule in left lower lobe shows multiple clusters of confluent punctate areas of calcification. Another pulmonary nodule with calcification is seen in right upper lobe in a subpleural location (arrow) (C and D).

On chest CT, multiple pulmonary soft tissue nodules were seen (Figures 6A, 6B). These findings were concerning for metastasis of chondroblastoma to the lung.

**Figure 7:**
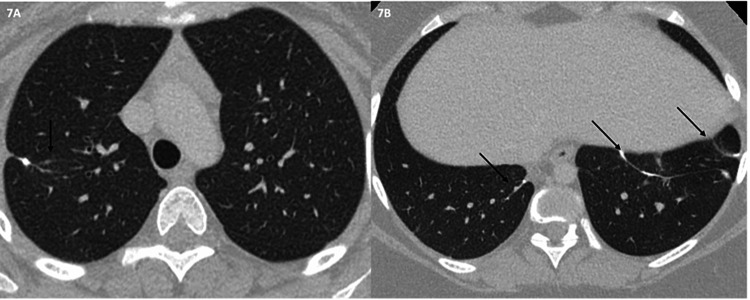
Chest CT in lung windows at upper and lower levels (A and B) at follow-up of five years, corresponding to levels shown in Figures 6A-6D. The image shows linear scarring and subpleural tags (postsurgical change) at sites of nodule resection (arrows). No new nodules are identified.

One month after detection of metastases on CT, the patient underwent robotic-assisted bilateral pulmonary wedge resections of seven nodules in the right lung and two nodules in the left lung. Microscopic review of the excised nodules demonstrated extensive peripheral ossification confirmed in all the foci of the tumor tissue. No significant atypia, atypical mitoses, or necrosis was identified. Immunohistochemical stain of the excised nodules for the H3K36M mutation found in chondroblastoma showed diffuse and strong immunoreactivity by mononuclear cells. Histology of the resected nodules resembled that of the primary tibial tumor. Therefore, final diagnosis of all resected lung nodules was consistent with chondroblastoma. Subsequently, the patient was started on denosumab 120 mg injection once a week for three weeks, then monthly for one year, then every three months indefinitely to prevent chondroblastoma recurrence. Most recent CT chest with IV contrast 14 months status post robotic-assisted bilateral pulmonary wedge resections showed no evidence of recurrent tumor (Figures 7A, 7B). No follow-up oncologic imaging of primary tumor site has been completed since the patient's last oncologic surgery 4.5 years ago. Treatment with denosumab is currently ongoing.

## Discussion

Metastatic chondroblastomas are very rare. The benign nature of chondroblastoma has been an issue of discussion. In their fifth edition of classification of bone and soft tissue tumors in 2020, the World Health Organization (WHO) re-classified chondroblastoma as a benign tumor, citing the prevalence of cases with metastases is less than 1% and therefore it should be considered benign. In the preceding fourth and third editions of the WHO classification of bone and soft tissue tumors, chondroblastoma was regarded as a neoplasm of intermediate malignancy, solely based on its ability to metastasize [3].

The mechanism of pulmonary metastases is hypothesized to be vascular invasion, possibly due to tumor embolization during surgical manipulation. However, there are cases of pulmonary metastasis of chondroblastoma before surgery discrediting this theory [4]. There is no clear explanation of the pathogenesis of lung metastasis in chondroblastoma [5]. It is well known that a point mutation, K36M, in the H3F3B histone protein-coding gene on chromosome 17 is present in approximately 95% of chondroblastomas [6]. However, a mutation in chondroblastoma that predicts metastatic or malignant potential has yet to be identified. Interestingly, A recent case of metastasizing chondroblastoma to the lung in which a next-generation sequencing (NGS) panel was performed demonstrated the presence of a monoallelic mutation in the MSH2 gene as well as a WHSC1L1-FGFR1 fusion transcript, which has otherwise only been reported in lung and breast cancer [3].

Radiographically, cases of pulmonary chondroblastoma are described to have variable presentation. They are reported to present on chest CT as nodules without calcification, as well as cystic, cavitary nodules [5,7]. The pulmonary metastases in our case presented as soft tissue nodules with punctate areas of calcification. Pulmonary metastases of chondroblastoma are reported to have been diagnosed incidentally on whole-body CT, on chest CT at original diagnosis, and on positron emission tomography (PET) CT to assess for metastasis [3,5,7].

In most cases of metastases of chondroblastoma, histological properties of the metastatic tumor are the same as those of the primary tumor. Pulmonary metastases have not been described to undergo malignant transformation and have not demonstrated histologic features of malignancy, such as hyperchromasia, atypia, or atypical mitoses [4]. Pulmonary metastases of chondroblastoma can cause serious morbidity and mortality. There is one published case in the literature of secondary pneumothorax caused by chondroblastoma. Some cases of pulmonary metastases have led to death [7]. There is scant discussion in the literature regarding management of pulmonary metastases of chondroblastoma due to their rare prevalence. One case of a primary chondroblastoma of the acromion with local recurrence and metastases to spine, soft tissue, and lung was treated with doxorubicin, followed by experimental treatment with pembrolizumab (due to a detected MSH2 mutation) with promising results. Of note, CT detecting pulmonary metastases and bone metastases demonstrated a decrease in metastases’ size following doxorubicin and pembrolizumab therapy [3]. Another case of chondroblastoma of the proximal left humerus with pulmonary metastases reports promising results with denosumab therapy following metastasectomy. The case reports calcification of pulmonary nodules and decreased size of nodules following 20 months of denosumab therapy [5]. To our knowledge, this is the only case in the literature that describes use of denosumab for chondroblastoma pulmonary metastases, other than our case.

## Conclusions

Chondroblastoma is a tumor of cartilage origin classified as a benign tumor by the WHO. Metastases of chondroblastoma, although rare, have been reported in literature, with the commonest site of metastatic involvement being the lung. There is no clear explanation in literature regarding the pathogenesis of metastatic disease in chondroblastoma. This case underlined the importance of suspecting metastatic disease in patients with a history of chondroblastoma when pulmonary nodules are detected on imaging. Practitioners should use this case as an example of small yet significant percentage of chondroblastoma cases that exhibit behavior that is not completely benign. The authors believe this would best be addressed by radiographic surveillance in select chondroblastoma cases, especially in those with known clinically aggressive behavior, such as our case. This would ensure optimal patient management in such cases.
